# The impact of age-related syndromes on ICU process and outcomes in very old patients

**DOI:** 10.1186/s13613-023-01160-7

**Published:** 2023-08-04

**Authors:** Hélène Vallet, Bertrand Guidet, Ariane Boumendil, Dylan W. De Lange, Susannah Leaver, Wojciech Szczeklik, Christian Jung, Sigal Sviri, Michael Beil, Hans Flaatten

**Affiliations:** 1 Institut National de la Santé et de la Recherche Médicale (INSERM), UMRS 1135, Centre d’immunologie et de Maladies Infectieuses (CIMI), Department of Geriatrics, Saint Antoine, Assistance Publique Hôpitaux de Paris (AP-HP), Sorbonne Université, F75012 Paris, France; 2 Institut Pierre Louis d’Epidémiologie et de Santé Publique, Hôpital Saint-Antoine, service de réanimation, Sorbonne Université, INSERM, AP-HP, 75012 Paris, France; 3 service de réanimation, AP-HP, Hôpital Saint-Antoine, F75012 Paris, France; 4 Department of Intensive Care Medicine, University Medical Center, University Utrecht, Utrecht, The Netherlands; 5 Department of Critical Care Medicine, St George’s Hospital London, London, England; 6 Intensive Care and Perioperative Medicine Division, Jagiellonian University Medical College, Kraków, Poland; 7 Division of Cardiology, Pulmonology and Vascular Medicine, University Hospital Düsseldorf, Heinrich-Heine-University, Düsseldorf, Germany; 8 Department of Medical Intensive Care, Faculty of Medicine, Hebrew University and Hadassah University Medical Center, Jerusalem, Israel; 9 Department of Clinical Medicine, Department of Research and Developement, Haukeland University Hospital, University of Bergen, Bergen, Norway

**Keywords:** Critical care, Intensive care unit, Old patients, Comprehensive geriatric assessment

## Abstract

In this narrative review, we describe the most important age-related “syndromes” found in the old ICU patients. The syndromes are frailty, comorbidity, cognitive decline, malnutrition, sarcopenia, loss of functional autonomy, immunosenescence and inflam-ageing. The underlying geriatric condition, together with the admission diagnosis and the acute severity contribute to the short-term, but also to the long-term prognosis. Besides mortality, functional status and quality of life are major outcome variables. The geriatric assessment is a key tool for long-term qualitative outcome, while immediate severity accounts for acute mortality. A poor functional baseline reduces the chances of a successful outcome following ICU. This review emphasises the importance of using a geriatric assessment and considering the older patient as a whole, rather than the acute illness in isolation, when making decisions regarding intensive care treatment.

**Supplementary Information:**

The online version contains supplementary material available at 10.1186/s13613-023-01160-7.

## Introduction

In the last decade, we have seen a steady rise in papers dealing with various conditions, aside from age, that are considered important in understanding the high mortality and morbidity associated with the critical ill old patient. While age is still an important factor, recent research has demonstrated that on its own, age only has a minor impact [1]. Hence, age should not be considered in isolation when making decisions about whether to admit a patient to the intensive care [2].

**Fig. 1:**
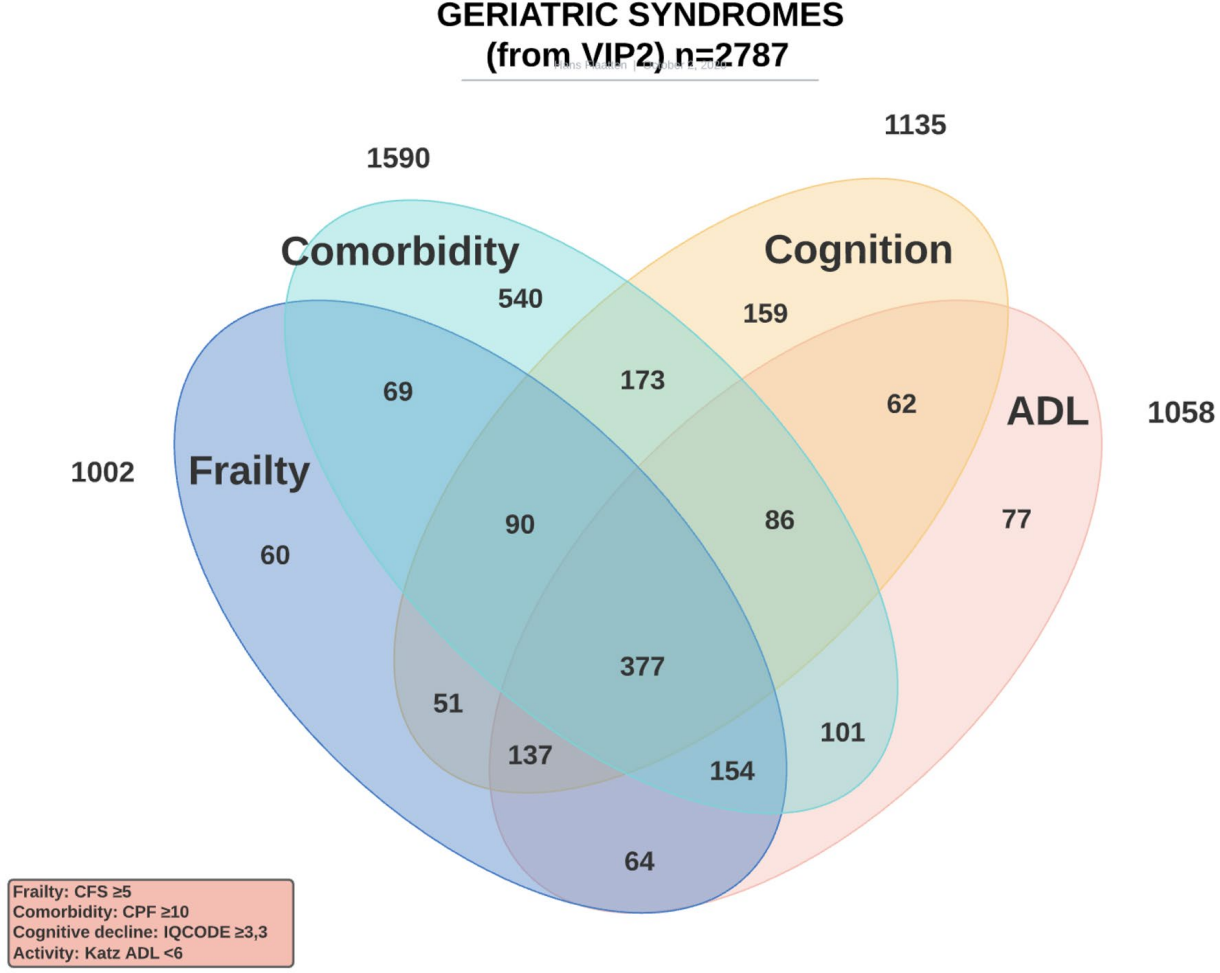
Overlap of four different geriatric syndromes in 2789 patients from the VIP2 study [3]

In this review, we aim to describe the current evidence for the use of ‘age-related syndromes’, a collection of different entities which overlap considerably (Fig. 1), when making decisions about the critically ill patient. Not all of these have a large impact alone [3] and we will describe in detail syndromes that it is now possible to evaluate prior to or on admission to intensive care unit (ICU) using simple methods. We will also comment on emerging issues for the very old ICU patients. It should be emphasised that the narrative review methodology has limitations and we have had to make choices.

## The impact of age

Age itself has an impact on short term survival, but also on the long term outcomes. In a large study from France using the French national health system database, adults (> 18 years) with an ICU admission between 2013 and 2015 were studied. Data from In-hospital and up to 3-year post-hospital discharge were analysed. They found a strong influence of age on survival from age ≥ 35 years, and in particular after the age of 80. The Odds Ratio (OR) of dying in hospital was more than eightfold higher in patients over 80 years and increased to more than 17 fold after the age of 90 [4]. They also found an impact on outcome from various co-morbid conditions and the reason for ICU admission, but frailty was not assessed in this registry study.

In the VIP2 study, age was not significantly associated with mortality in octo- and nonagenarians when corrected for confounders, such as frailty, comorbidity, cognition and comorbidity (3), but this does not rule out an effect if compared with younger ICU patients.

## The impact of physiological aging, focus on immunosenescence

Over time the immune system undergoes several alterations called immunosenescence. The phenotype but also the function of cells and organs change.

### Aging of innate and adaptive compartment and inflammaging

The consequences of aging on the innate compartment are essentially functional alterations, the number of cells is not impacted. In neutrophils, the capacity of phagocytosis, chemotaxis and oxidative stress residue production are decreased, while their clearance is increased [5, 6]. The subtype distribution of monocytes changes in favour of pro-inflammatory intermediate (CD14 + /CD16 +) and unconventional (CD14-/CD16 +) monocytes [7]. Their abilities of migration and adhesion are impacted [8, 9]. Furthermore, phagocytosis of monocytes and dendritic cells and antigen presentation capacities of dendritic cells are altered [10, 11].

Aging of the T cells is characterised by 3 main types of changes: 1. the decrease of naive T cells number due to thymic involution [12]; 2. the shrinking of the T Cell Receptor (TCR) repertoire and thus of the capacity to recognise neo-antigen [13]; 3. the increased proportion of terminally differentiated oligoclonal memory T cells [14, 15]. There is also a more pronounced regulatory profile during aging with an increased population of regulatory TLs (CD4 + CD25 +), a decreased capacity to produce IFN-g and an increased expression of several immune checkpoint inhibitors (CTLA4, PD-1 and LAG-3) [13, 16, 17]. The total B cells pool decreases [18], with an accumulation of exhausted memory B cells and an impairment of class switch recombination [19]. The consequences are a decrease of antibody production [20] and an impaired production of higher affinity protective antibody [21].

Aging is associated with a chronic low-grade systemic inflammatory state called “inflamm-aging” [22]. It is characterised by the increased production of pro-inflammatory cytokines, such as IL-1β, IL-6 and TNF-ɑ [23]. Mechanisms involved in this proinflammatory state are multiple: oxidative stress [24], persistent DNA damage [25], stem cell aging [26], inhibition of autophagy by activation of inflammasome [27]. The level of this pro-inflammatory state is associated with a worse prognosis in older patients: increased morbidity, mortality, sarcopenia and frailty [23]

### 3.2 Clinical consequences of immunosenescence

All these changes and their interaction have numerous clinical consequences, such as frailty, sepsis or cancer.

Interestingly, frail people present an immune profile more senescent than robust people with a higher rate of terminally differentiated CD8 + CD28-T cells [28, 29] and a lower IL-17 production after in vitro stimulation [29].

Thus, old patients are at greater risk of developing septic shock and mortality is higher than in the younger population [30]. Viral infections such as influenza or Varicella Zoster Virus (VZV) are also more frequent and more severe [31, 32]. Vaccine efficacy is reduced in the old patients, in particular because of the lack of priming of T-cells and the reduced capacity of plasma cells to produce antibodies [33, 34]. Finally, the shrinking of TCR, the decrease in the cytotoxic capacities of senescent T-cells and the decrease in their migration capacity lead to a reduction in tumour control by the immune system and an increase in the incidence of cancers over time [35].

There is currently no biomarker, feasible in routine biology, that is specific for immunosenescence. However, we can note the decrease in the total lymphocyte count [36], the inversion of the CD4 + /CD8 + ratio [37], the decrease in IL-10 levels [38] or the increase in IL-6 and CRP levels [39, 40]. The processes underlying Immunosenescence and inflammaging are complex. The future probably lies in the development of personalized immunological monitoring, which can be performed at the patient’s bed.

## The impact of frailty

Frailty is a condition characterised by the loss of biological reserves, failure of homeostatic mechanisms and vulnerability to a range of adverse outcomes, such as falls, disability, hospitalisation, cognitive decline and the need for care [41]. The concept has been used within geriatrics for decades, but is now slowly being adopted by many other specialities.

Frailty is probably the most studied “syndrome” in the very old. Within intensive care it has gained popularity since its introduction in 2014 [42]. The number of studies using frailty as the main or secondary objective is increasing.

With unpublished detailed data from the VIP2 study [3], we show in Fig. 1 that the critically ill old patient usually has more than one syndrome, but it is interesting that frailty is the syndrome that is most frequently present with one of the other syndromes, in particular with cognitive decline, reduced activities of daily living and co-morbidity. Frailty was found as an isolated syndrome (with no other disability) in only 6% of patients, and reduced activity of daily living (ADL) was found similarly seldom alone (7%). Co-existence of frailty and ADL was found in 56% of patients and 732 of 1328 patients had either frailty or reduced ADL.

It is not possible to measure frailty using a simple objective test. Traditionally an in-depth comprehensive geriatric assessment (CGA) has been used to identify frailty [43] and could be considered the gold standard tool to identify and assess it. However, since this requires time and usually a team of assessors, including a geriatrician, such an assessment is beyond reach in acute and urgent situations, such as prior to an ICU admission. For this reason, several alternative means to assess frailty have evolved [44]. Of these, two have been used regularly in ICU patients: the Clinical Frailty Scale (CFS) [45] and the modified Frailty Index (mFI) [46]. They are very different. The CFS is a visual scale that is composed of 9 classes from very fit to terminally ill (Additional file Figure). Whereas the mFI uses several states correlated with frailty, usually derived from diagnostic codes, such as the International Classification of Diseases (ICD), and has been used to extract data from clinical databases without the need to see the patient. From its original version, the list has grown shorter and today only include 5 items [46] and, therefore, at best provides only a very superficial screening tool for documenting frailty (Additional file 1: Table S1).

Frailty has consistently been found to have a great influence on ICU survival in old ICU patients. A systematic review [47] confirmed this and found that on average 40% of patients above 80 were frail. In the VIP2 study [3] frailty was found to provide better prognostic information than age, organ failure and cognition. However, frailty alone does not have enough sensitivity and specificity to be able to predict survival in the individual patient.

Patients with frailty have also been found to receive less vital organ support during ICU admission (mechanical ventilation and use of vasoactive medication) with an increased ICU length of stay. [48] This was less pronounced in the more recent VIP1 and VIP2 studies (Table 1), where the picture was less convincing. However, the VIP studies included only patients  ≥ 80 years [1, 3] and hence differs from the former study.

**Table 1:** Use of ICU procedures and ICU LOS in three ranges of CFS in two large prospective European studies (VIP)

CFS class	*n* =	Mechanical ventilation	%	Vasoactive drugs	%	RRT	%	LOS (days)
< 4	3493	1851	53.0	1879	53.8	887	25.4	5.7
4	1792	887	49.5	973	54.3	199	11.1	6
> 4	3799	1884	49.6	2198	57.9	411	10.8	5.7

Data extracted from VIP1 (Flaatten H et al. Int Care Med 2017 [1]) and VIP2 (Guidet B et al. Int Care Med [2]) studies

*CFS*   Clinical frailty score; *LOS*   Length of stay; *RRT*  Renal replacement Therapy)

A particular concern is the connection between frailty and persistent critical illness (PCI), also called chronic critical illness. This is a term used for ICU long-stayers and a length of stay of more than 10 days has often been used, but definitions vary [49]. In a recent population study from Australia and New Zealand it was found that 3.3% of all patients admitted to the ICU developed PCI, and after 10 days the severity of illness was no longer more predictive for mortality than pre-hospital characteristics. Frailty was found to be associated with both developing PCI and death [50].

## The impact of comorbidities

Comprehensive geriatric assessment is a global approach used by geriatricians. It encompasses several dimensions including comorbidities. The proportion of patients with comorbidities and the number of comorbidities per patients increases with age. The mean number of comorbidities per patients is 2.6 ± 2.2 in patients 65 to 84 years and 3.6 ± 2.3 in patients 85 years or over [51]. The most common comorbidities are hypertension, diabetes, chronic obstructive pulmonary disease, cardiac failure, cancer and cognitive impairment [52]. Comorbidities are associated with an increased mortality [53], this is also seen in ICU patients [54]. Several tools have been used to assess comorbidities. Composite scores relying on ICD-9 or ICD-10 codes have been developed, such as the Charlson Comorbidity index (CCI) [55]. The score assesses the number but also the severity of comorbidities. The CCI has been validated in critically ill patients and is predictive of mortality [55, 56]. However, for ICU or trauma patients, the CCI did not perform as well as other instruments to predict prognosis [57]. Other scores simply count the number of comorbidities, such as the Comorbidity and polypharmacy score [58].

As presented in Fig. 1, at admission comorbidities might be present alone. In our VIP2 study, comorbidities were not an independent factor for predicting 1-month mortality [3]

The contribution of comorbidities to the prognosis and treatment strategy has been tested in several patient categories. This outlines the absolute necessity to assess a patient globally to individualise the treatment. More and more old patients will be admitted to ICU with chronic disease.

Old patients with malignant haematologic diseases are increasing and several may require ICU admission. Since the prevalence of comorbidities increases with age, it is important to assess the contribution of comorbidities to the prognosis.

Multiple myeloma (MM) is a disease which commonly occurs in older patients. Novel agents have allowed a major improvement in outcome. Old patients are more susceptible to side effects and often require lower dose intensity regimens. Identification of vulnerable patients through geriatric assessment including comorbidities enables optimisation of treatment and ultimately survival of older patients with myeloma [59].

Chronic lymphocytic leukemia (CLL) is also a common haematologic malignancy in old patients. Recent work has included the use of geriatric assessment, Charlson comorbidity index, cumulative illness rating scale, and most recently, the CLL-comorbidity index for choosing the best treatment regimen [60].

The incidence of acute myeloid leukemia increases with age, and more than half of AML patients are over 60 years. A poorer prognosis in old patients is related to age, functional status, and comorbidities, combined with leukemia characteristics. Screening of candidates for aggressive treatment relies on patient characteristics (geriatric assessment including comorbidities) and disease characteristics (cytogenetics and molecular parameters) [61].

The incidence of solid tumor increases also with age. The Eastern Cooperative Oncology Group Performance Status (ECOG PS) score is widely used by oncologist. This score was developed 40 years ago as an adaption of the 70-year-old Karnofsky performance score. It is a unidimensional functional and subjective score. It fails to account for multimorbidity, frailty or cognition. In a recent position paper, a strong recommendation is made to move to routine use of the CFS to help to triage patients and to design appropriate treatment and rehabilitation interventions [62].

The contribution of comorbidities to prognosis has also been estimated in surgical patients, such as for total hip arthroplasty [63] liver transplant [64], head and neck free flaps [65] and for trauma patients [66]. In all these publications it is recommended that age per se should not be used for choosing treatment strategies. It is difficult to sort out the impact of comorbidities from other geriatric variables.

### Focus on cognition

Cognitive decline is prevalent among old patients, but there is a wide spectrum of dementia. We have found in a previous study that dementia was not an independent prognosis factor for 6-month survival among patients aged 80 years and over admitted to ICU. Two factors often associated with dementia were identified: functional decline assessed with ADL and nutritional status [67]. This outlines the need to assess the consequences of dementia and not merely use a cognitive tool. Most of the tools used to measure cognitive decline are not easy to use in urgent situations. A Mini Mental-State Examination (MMSE) has been developed to help overcome this difficulty. In VIP2 study, we used the IQcode [68] which is a 16 items questionnaire; with IQCODE ≥ 3.5 defining cognitive decline. This questionnaire relies on caregivers. Among 3913 patients, IQcode was not measured, even if caregivers were present, in 334 patients (8.5%) and it was not measured in 599 patients, because there was no caregiver present (15.3%). The high percentage of missing information suggests that such information is difficult to collect. Patients ‘ characteristics were different, with more frail patients, a decrease in functional status, a higher severity assessed with Sepsis-related Organ Failure Assessment (SOFA) and a higher in ICU mortality seen in those when this information on IQcode was missing. Moreover, in multivariate analysis IQcode was not an independent prognosis factor at 1 month [3]

### Focus on sarcopenia

Sarcopenia is considered one of the major “geriatric syndromes” and may have a major impact in the old ICU patient. Sarcopenia is the progressive, often age induced, decline in muscle function and is described as a combination of loss of both muscle mass and muscle function. Several methods are used to assess sarcopenia. Hand-dynamometry is frequently used to measure muscle strength (age and gender corrected) and ultrasound may be used at the bedside to assess muscle mass. Obviously, in the critical ill old patients the number of methods available to assess muscle strength are reduced due the inability to co-operate, but evaluation of muscle mass is feasible using different methods.

For intensivists used to ultrasound, this method is probably the method of choice as the equipment is familiar [69]. However, a recent metanalysis has documented good correlation between DXA and CT assessment of rectus femoris or gastrocnemius muscles [70].

An increasing number of studies have documented the independent negative effects of sarcopenia on outcomes after intensive care in general and in old patients in particular [71].

Another emerging issue is the concept of acute muscle wasting disorder connected to bedrest and inactivity which is more pronounced during hospitalisation [72]. This has been demonstrated in healthy volunteers and is more pronounced in older adults [73]. A recent study in patients with intra-abdominal sepsis demonstrated persistent loss of muscle mass after hospitalisation both in patients with and without preadmission sarcopenia [74]. In that study pre-admission sarcopenia was independently associated with 6-month survival in contrast to persistent muscle mass disorder, highlighting that the interaction between chronic and acute sarcopenia during critical illness is complicated and far from fully understood.

### Focus on malnutrition

The prevalence of malnutrition in old critical patients varies from 20 to 60% depending on the assessment method used [75, 76]. Several factors contribute to malnutrition: low food intake, monotonous diets, swallowing disorders and reduced intestinal absorption. The chronic nutritional state should be assessed before high-risk surgery and also as soon as possible after ICU admission. On top of baseline malnutrition, acute stressors, such as sepsis, trauma, pancreatitis adds to the catabolic state.

Fat mass increases steadily with age, while lean mass decreases. Muscle mass falls by 3–8% per decade from age 30 and declines faster after the age of 60, with a predominant reduction in the number of type II fibers (with rapid contraction and glycolytic metabolism). Insulin resistance, promoted by low physical activity and low-grade inflammation, is accompanied by a decrease in the muscular capacity to oxidise fatty acids and to use glucose. This anabolic resistance justifies a high daily protein intake, although direct evidence is lacking for ICU old patients.

Malnutrition in critically ill patients is associated with an increased risk of infection, extended length of stay and may lead to poor quality of life, disability, and morbidity long after ICU discharge [77, 78]. It has been identified as an independent prognostic factor 6 months after ICU discharge [67].

Assessing nutritional status is not an easy task. It should be stressed that Body Mass Index (BMI) is often falsely reassuring due to fluid retention increasing weight and a reduction in height with aging. The WHO use a cutoff value of 20 kg/m^2^ to define malnutrition. Until a specific tool has been validated, the European Society for Clinical Nutrition and Metabolism recommends the use of anamnesis (weight loss or recent decrease in physical performance), physical examination, general assessment of body composition, and muscle mass and strength [77].

Vitamin D insufficiency is the most common nutritional deficiency, occurring in 40–60% of the healthy general adult population and is frequently seen in ICU patients (60–95% Vitamin D deficient or insufficient) of any age [79]. Vitamin D deficiency (various cutoff between 11 and 25 ng/ml) could cause complications, especially infectious and may lead to an increased length of stay. Low transthyretin (TTR also known as prealbumin) levels at ICU admission are independently associated with a higher in-hospital mortality, more infectious complications, longer total hospital length of stay (LOS), and ICU LOS [80]. According to their prognostic performance, albumin and prealbumin levels remain the most widely used “nutritional” biological markers [81]. TTR and Retinol-Binding Protein (RBP), as rapid turnover proteins, can be used to monitor nutritional therapy lasting more than a week and are correlated with energy intake and nitrogen balance in ICU. Efficient and accessible biomarkers for nutritional risk or the efficacy of a nutritional intervention in old ICU patients have to date not been identified [82].

Where possible oral diet and oral nutritional supplements remain the first-line intervention for the non-ventilated ICU patient [77]. However, energy intake is likely to be suboptimal in old ICU patients, due to decreased appetite, alterations in taste and smell, gastrointestinal symptoms, weakness, delirium, or abulia. Dietary monitoring is essential to assess inadequate oral intakes and decide without delay when to implement artificial nutrition. Particular attention should be paid to screening for swallowing disorders that are frequently present in the old population during an acute illness.

If oral intake is not possible or insufficient, ESPEN guidelines on clinical nutrition in ICU recommend nutritional support within 48 h for enteral nutrition (EN) or within 3–7 days for parenteral nutrition (PN) in cases, where enteral nutrition is not possible [77]. In a meta‐analysis comparing enteral versus parenteral feeding strategy in ICU, the enteral route was not associated with a significantly reduced overall mortality, but reduced rates of ICU‐induced infection and length of stay [83].Most studies highlight the deleterious effects of a high-calorie intake with possible refeeding syndrome.

Physical activity is recommended to improve the beneficial effects of nutritional therapy [77].

Among ICU survivors, early exercise training (passive or active cycling) enhanced recovery of functional exercise capacity and muscle force at hospital discharge [84]. However, a Cochrane review, analyzing early intervention (mobilization or active exercise) starting in the ICU, concluded that the evidence for benefit was poor for physical function or performance, muscle strength, or health‐related quality of life [85]. In a recent RCT testing the impact of increased early mobilisation (sedation minimisation and daily physiotherapy) on 750 invasively ventilated adult ICU patients, there was no difference found in the number of days that patients were alive and out of the hospital. Furthermore, the intervention was associated with an increased number of adverse events [86].

## The impact of functional status

Loss of functional autonomy is probably one of the more frequent complications occurring in frail old patients after an acute stress. The “Activity of Daily Living” scale (ADL) was used in the VIP2 study and is a simple instrument that can be used at admission to score the activity level prior to the acute illness. Provided the patient does not have severe cognitive dysfunction, it has been shown to correlate well between patients and a proxy on admission [87]. Frailty and loss of autonomy are frequently found simultaneously. In Fig. 1 with data from the VIP2 study, we can see that only 27% of frail patients have no reduction in ADL. In this study it was also shown to significantly contribute to the observed mortality in critically ill old patients [3]. A similar finding was recently reported in old ICU patients with COVID-19 [88]. The functional status of 754 old people living in the community was followed over a 4-year period. 259 were eventually admitted to an ICU. The most important factor influencing 1-year post-ICU mortality was a downward trajectory to severe disability prior to ICU admission. This effect was greater than that seen from mechanical ventilation and shock [89].

## The impact of admission diagnosis

Besides, geriatric assessment and age, reason for ICU admission has a profound impact on short-term prognosis but also on long-term outcome. Mortality is much lower among patients admitted to ICU after scheduled surgery compared to patient requiring urgent surgery [90]. For medical patients, the patient and ICU stay characteristics are very different according to admission diagnosis (Table 2) [3]. For example, the outcome is much worse for neurological patients than for other admission categories.

**Table 2:** Organ supports and ICU stays characteristics according to reason for ICU admission (VIP2 data)

		Circulatory failure	Combined respiratory/circulatory failure	Emergency surgery	Multitrauma w/wo head injury	Multitrauma without head injury	Other	Respiratory failure	Sepsis (according to Sepsis3)	*p*-value
Invasive MV	yes	247 (45.7%)	307 (68.4%)	307 (56.9%)	54 (72%)	36 (41.4%)	321 (43.2%)	417 (44.3%)	264 (49.2%)	< 0.0001
Duration IMV (hours)	med (IQR)	42 (16–96)	72.5 (29.2–177.5)	24 (12–95.5)	120 ( 24.5–245.5)	91.5(17.75–210.75)	46 (14–160.25)	111.5 (36–240)	75 (24–231)	< 0.0001
NIV	yes	67 (12.4%)	135 (30.1%)	64 (11.8%)	5 (6.8%)	17 (19.5%)	48 (6.5%)	481 (51.2%)	86 (16%)	< 0.0001
Duration NIV (hours)	med (IQR)	12 (4–32.5)	21 (6–50.5)	24 ( 8–44.5)	22 (20–88)	12.5 (7.5–35.7)	20 (7.7–39)	20 (6–50)	16 ( 4–56)	0.2401
Tracheostomy	yes	19 (3.5%)	41 (9.2%)	20 (3.7%)	9 (12%)	9 (10.3%)	46 (6.2%)	85 (9%)	35 (6.5%)	< 0.0001
Vasoactive drugs	yes	372 (68.8%)	342 (76.3%)	363 (67.2%)	51 (68%)	43 (49.4%)	289 (38.9%)	409 (43.4%)	460 (85.5%)	< 0.0001
Duration vaso active drugs(hours)	med (IQR)	32 ( 14–69.25)	52 (20–121)	35 ( 16–72)	58(18–109.5)	45 (24–116)	35 (13–96)	60 ( 24–144)	48 (22–110)	< 0.0001
Renal replacement therapy	yes	50 (9.2%)	73 (16.3%)	40 (7.4%)	6 (8.1%)	8 (9.2%)	67 (9%)	75 (8%)	110 (20.5%)	< 0.0001
Withholding LST	yes	165 (30.7%)	142 (32.2%)	123 (22.9%)	21 (28.8%)	20 (23%)	166 (22.6%)	314 (33.6%)	189 (35.7%)	< 0.0001
Delay admission withholding decision	med (IQR)	1 (1–2)	2 (1–4)	2 (1–4)	2 (1–6)	2 (1–4.5)	1 (1–3)	1 (1–4)	1 (IQR 1–4)	0.0349
Withdrawing LST	yes	90 (16.7%)	87 (19.7%)	55 (10.2%)	11 (14.9%)	9 (10.3%)	87 (11.8%)	125 (13.4%)	81 (15.3%)	< 0.0001
Delay admission withdrawing decision	med (IQR)	3 (2–4)	4(2–6)	4(2–8)	5 (2–9)	9 (2–20)	3 (1–6)	5 (2–8)	3 (2–8)	< 0.0001
ICU LOS for non-survivors	med (IQR)	3 (1.5–5.4)	4.06 (1.9–8.0)	3 (1.5–6.7)	6 (1.9–13.0)	4.7 (2.2–9.0)	3 (1.4–6.5)	5 (2.2–9.9)	4.75 (2–9.0)	< 0.0001
ICU LOS for survivors	med (IQR)	3 (1.7–5.8)	4.4 (2–8.6)	2.9 (1.5–5.0)	4.8 (1.9–11.9)	4.44 (2.0–6.9)	3 (1.6–6.0)	4.75 (2.2–9.0)	5 (2.5–8.8)	< 0.0001
ICU mortality	*n* (%)	151 (28%)	208 (46.8%)	110 (20.4%)	26 (34.7%)	21 (24.1%)	145 (19.6%)	242 (25.8%)	169 (31.5%)	< 0.0001

*MV* mechanical ventilation; *NIV* non invasive mechanical ventilation; *RRT* renal replacement therapy: *LST* life sustaining treatments; *LOS* length of stay

## Outcomes

For decades intensivists have been concerned about the seemingly poor outcome of old ICU patients. In a study from 1998, including 6243 ICU patients from the US, the ICU admission rate was higher in patients above the age of 60 when compared with those below 60 (60% versus 30%, respectively), while mortality, length of stay and charge per day were higher in the older population. This led to questions regarding the utility of ICU for older patients in whom the expected outcome is poor and the cost high [91]. Between 1992 and 1996, the mortality decreased from 38 to 30% in those < 60 years of age but increased from 62 to 70% in patients above 60 years. In the same time period, a study from France of ICU patients above 75 years, could not find very old age to be directly associated with ICU mortality [92]. Some years later, the outcome in 233 octogenarians (≥ 80 years) in a single centre medical ICU from Paris was studied. The long-term survival at 2 m, 2y and 3y after admission were 59%, 33% and 29%, respectively [93]. Their analyses revealed two important factors for a poor outcome: an underlying fatal disease and severe functional limitation. If neither of these were present, the remaining patients had a reasonably good quality of life.

Octogenarians have subsequently been the focus of many outcome studies in intensive care patients. This is probably because the perceived benefit of such health care in this group is of particular interest, but also as it will be one of the fastest growing demographic groups in the next few decades.

Survival is the most frequently measured outcome, and this topic was recently highlighted in a systematic review [94] Most studies have been performed in the USA and Europe and the review focused on studies conducted after the year 2000. The study found a substantial variation in both short and long term survival, probably due to the large heterogeneity and size of the studies included. In addition, there were differences between retro and prospective studies and between single versus multicentre studies. In most of the larger studies ICU mortality was found to be between 20 and 30%, and hospital mortality 30–40%. There are few data on long-term outcomes, but in the ten studies found in general ICU patients, the 6-month mortality ranged from 21 to 58%.

Patient reported outcomes are less frequently studied. A Canadian study from 2015 found that half of the 1-year survivors (25% of the study population) had an acceptable quality of life [95]. In a large population (> 3000) of old ICU patients with COVID-19; 3-month survival was 39% and nearly half of the patients had severe or extreme problems with at least one item in the EurQol-5D-5L questionnaire [96]. Such data are important and the current knowledge of non-mortality data in the very old ICU survivors must be expanded. With the current picture of only 50% surviving to 12 months and half of survivors having a poor quality of life, we really need to continue the search for reliable prognostic factors, present at admission, to be able to deliver intensive care to those with a high probability of benefiting from such intervention.

## Conclusion

In elderly patients, several intricate factors contribute to the decision to admit to the ICU, ICU treatment intensity during the ICU stay and short- and long-term quantitative (mortality) and qualitative (HRQOL, Functional status) outcomes. It is important to emphasise that in making these decisions, age alone is less important than underlying geriatric conditions suggesting that geriatric tools should be used routinely during the whole patient trajectory from before ICU admission to after ICU discharge (Additional file 1: Table S2).

### Supplementary Information

**Additional file 1: Figure S1.** Clinical Frailty Scale by Rockwood. **Table S1.** The modified 5-item Frailty Index (mFI). **Table S2.** A summary of important tests and examination to be performed at/during admission in the critical ill elderly patients.

## Data Availability

All data and materials may be requested to the corresponding author: Pr B Guidet (bertrand.guidet@aphp.fr).
