# Supplementary material for: The impact of age-related syndromes on ICU process and outcomes in very old patients
Source: Ann Intensive Care. 2023 Aug 4;13:68. doi: 10.1186/s13613-023-01160-7 (PMC10403479; doi:10.1186/s13613-023-01160-7)
Supplement: Supplementary file 1 — Additional file 1: Figure S1. Clinical Frailty Scale by Rockwood. Table S1. The modified 5-item Frailty Index (mFI). Table S2. A summary of important tests and examination to be performed at/during admission in the critical ill elderly patients. [file 13613_2023_1160_MOESM1_ESM.docx]

Additional data

**Figure S1: Clinical Frailty Scale by Rockwood**


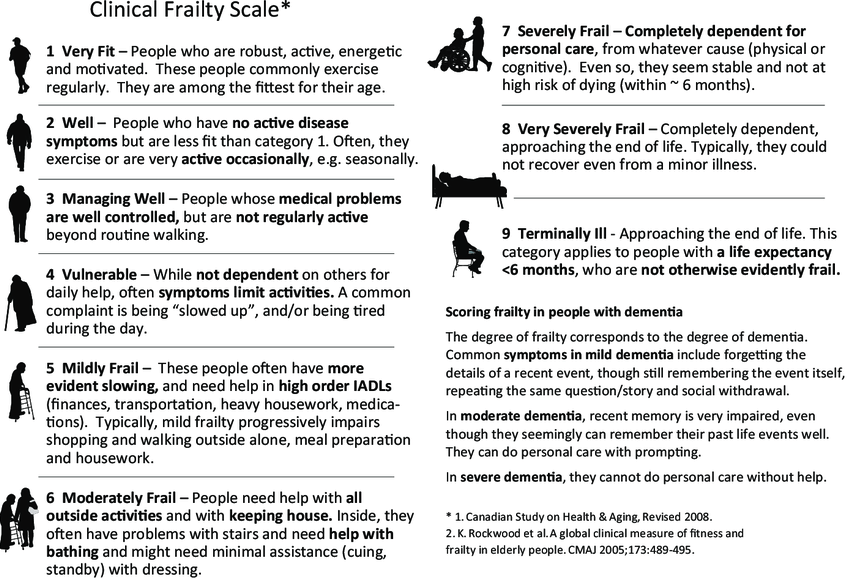


**Table S1 : The modified 5-item Frailty Index (mFI)**

| Factors included in mFI-5 | |
| --- | --- |
| 1 | Functional health status before surgery (either partially or totally dependent) |
| 2 | Diabetes mellitus (noninsulin or insulin) |
| 3 | History of COPD |
| 4 | Congestive heart failure within 30 days before surgery |
| 5 | Hypertension requiring medication |

**Table S2. A summary of important tests and examination to be performed at/during admission in the critical ill elderly patients.**

| **Testing** | **Test/examination** |
| --- | --- |
| Frailty | Clinical Frailty Scale |
| Polypharmacy | Summary of daily prescription drug used |
| Co-morbidity | Charlson Comorbidity Score |
| Malnutrition | Body Mass Index |
| Functional status | Activity of daily life (ADL) |
| Muscle strength/mass | Hand Grip Dynamometry (if co-operable). Ultrasound: rectus femoris |
